# Soft Electromagnetic Vibrotactile Actuators with Integrated Vibration Amplitude Sensing

**DOI:** 10.1021/acsami.3c05045

**Published:** 2023-06-16

**Authors:** Mert Vural, Mohsen Mohammadi, Laura Seufert, Shaobo Han, Xavier Crispin, Anders Fridberger, Magnus Berggren, Klas Tybrandt

**Affiliations:** † Laboratory of Organic Electronics, Department of Science and Technology, Linköping University, 602 21 Norrköping, Sweden; ‡ Wallenberg Wood Science Center, ITN, Linköping University, 602 21 Norrköping, Sweden; § Department of Biomedical and Clinical Sciences, Linköping University, 581 83 Linköping, Sweden

**Keywords:** stretchable electronics, soft actuators, electromagnetic actuators, cellulose nanofibrils, strain sensors

## Abstract

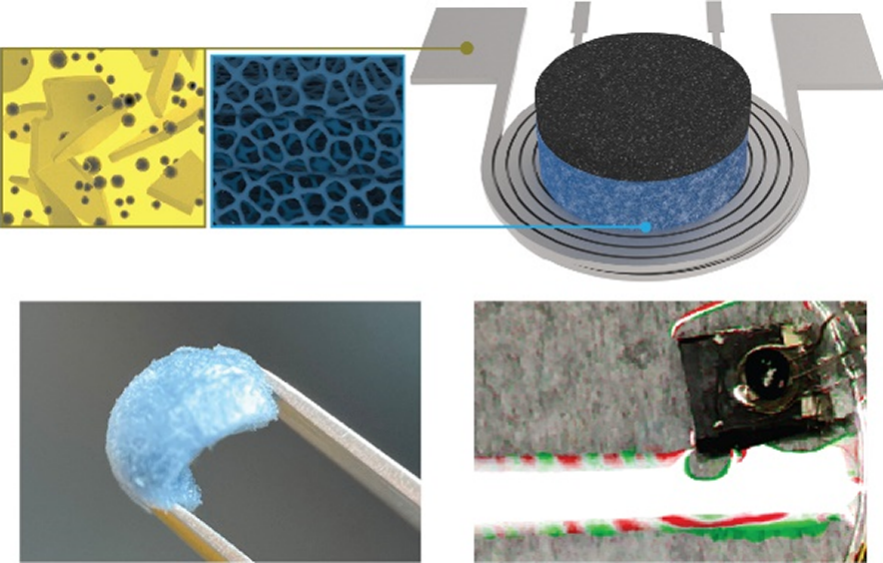

Soft vibrotactile devices have the potential to expand the functionality of emerging electronic skin technologies. However, those devices often lack the necessary overall performance, sensing-actuation feedback and control, and mechanical compliance for seamless integration on the skin. Here, we present soft haptic electromagnetic actuators that consist of intrinsically stretchable conductors, pressure-sensitive conductive foams, and soft magnetic composites. To minimize joule heating, high-performance stretchable composite conductors are developed based on in situ-grown silver nanoparticles formed within the silver flake framework. The conductors are laser-patterned to form soft and densely packed coils to further minimize heating. Soft pressure-sensitive conducting polymer-cellulose foams are developed and integrated to tune the resonance frequency and to provide internal resonator amplitude sensing in the resonators. The above components together with a soft magnet are assembled into soft vibrotactile devices providing high-performance actuation combined with amplitude sensing. We believe that soft haptic devices will be an essential component in future developments of multifunctional electronic skin for future human–computer and human–robotic interfaces.

## 1 Introduction

Research to generate artificial perception for human senses via electronics has resulted in devices with the ability to imitate and perceive sound, visuals, and even the sense of touch, which has found many applications in biomedicine and virtual and augmented reality.^1−10^ Current electronic technologies can sense and mimic sounds and visuals with utter clarity, yet haptic systems targeting touch are still considered primitive.^4−8,11^ Several concepts including electrostimulation of muscles,^12,13^ vibrotactile actuation generated via pneumatic actuators,^3,7,14^ piezoelectric materials,^15,16^ and electrical motors^5^ are used to construct haptic systems that can initiate spatially resolved vibrotactile responses on the skin. Spatial and temporal variation in electrical properties of epidermal tissue across skin makes it difficult to find an optimal and stable voltage or current setting to initiate a consistent tactile response for electrostimulation.^12,13,17,18^ This problem also engenders additional difficulties including alterations in the surface between epidermal tissue and electrodes, which leads to patient discomfort, and inflammation on the skin.^12,13^ Haptic systems based on soft pneumatic actuators prove to be a better alternative for stimulating the sense of touch, due to their consistent tactile response.^3,7,14^ In addition, soft pneumatic actuators form conformal interfaces with the skin, since they are mostly constructed from elastomers that match the mechanical properties of the epidermal tissue.^3,7,14^ However, soft pneumatic actuators have operation frequencies limited to below 100 Hz,^5^ which excludes the reception frequency of several mechanoreceptors in epidermal tissue.^19^ Moreover, these actuators are more susceptible to mechanical failure as they require the entire actuator system to be tightly sealed.^5^

Haptic systems consisting of piezoelectric materials and electric motors can provide a consistent and scalable mechanical response at frequencies far exceeding the sensitivity limits of the epidermal tissue.^5,15,16^ Nevertheless, these systems usually consist of bulk devices that are applied to the skin using mounting substrates consisting of textiles with elastomer films to properly interface these large devices with the epidermal tissue.^5^ There have been recent efforts on the development of the soft vibrotactile haptic systems, particularly systems based on electromagnetic actuators.^4,11^ These devices either consist of geometrically deformable circuits constructed on elastomer substrates^4^ or circuits constructed using intrinsically deformable conductors that are embedded in elastomer substrates.^11^ In either case, several components of the devices are still composed of brittle and bulky materials. A major challenge when developing soft electromagnetic devices is the need for low-resistance coils, which require both highly conductive stretchable composites and high-aspect-ratio structures. Composites based on silver flakes (AgF) constitute one of the most promising systems for high-performance stretchable bulk conductors.^20^ The incorporation of silver nanoparticles (AgNPs) formed from AgF inside the matrix can improve conductivity and stability under high strains by bridging the gaps between AgFs and thereby improving the electrical contact between the flakes.^21−25^ As an alternative, nanoparticles can be prepared and mixed during the composite fabrication process.^26,27^ Yet, another way is by forming AgNPs in situ by chemical reduction of silver ions, from which it is possible to obtain high concentrations of well-dispersed AgNP within a polymer matrix,^28−34^ but this method has so far not been used together with AgFs and thus the optimal amount of AgNP is unknown. A compact soft haptic system entirely based on soft materials and composites with mechanical properties analogous to epidermal tissue is yet to be demonstrated, which can provide new opportunities in feedback systems for prosthetics, virtual and augment reality tools.

**Figure 1:**
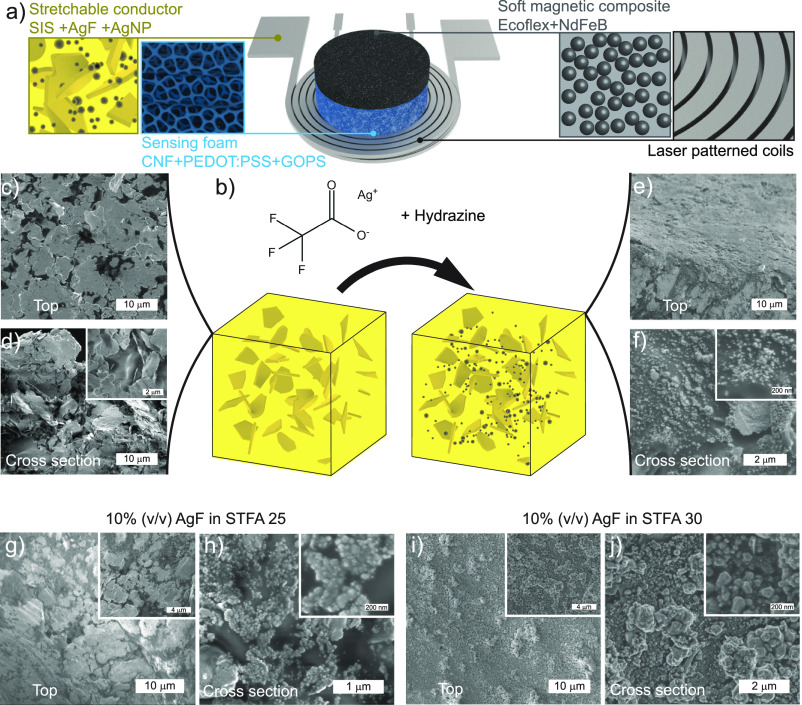
Actuator design and stretchable AgF/AgNP conductors. (a) Schematic of the vibrotactile device and its components. (b) Schematic illustration of stretchable conductors consisting of Ag microflakes (AgFs), poly(styrene-block-isoprene-block-styrene) (SIS) block copolymer, and Ag nanoparticles (AgNPs). (c) SEM top view and (d) cross-section images of AgFs/SIS composites. (e) SEM top view and (f) cross-section images of stretchable conductors with 10% (v/v) AgFs incubated in STFA 20 (0.2 g/mL). (g) SEM top view and (h) cross-section images of stretchable electrodes with 10% (v/v) AgFs incubated in STFA 25. (i) SEM top view and (j) cross-section images of stretchable electrodes with 10% (v/v) AgFs incubated in STFA 30.

Here, we present a soft haptic system based on soft electromagnetic actuators that consists of intrinsically stretchable conductors, pressure-sensitive conductive foams, and stretchable magnetic composites (Figure 1a). We address the need for high conductivity stretchable conductors by developing a method for enhancing the conductivity in silver flake composites by in situ nanoparticle growth. High-resolution high-aspect-ratio coils are fabricated by laser cutting and integrated into the soft actuators. Through the incorporation of strain-sensitive soft cellulose-conducting polymer foams, the actuators can internally sense the actuation amplitude, which can be useful for feedback control of the amplitude in systems with strong resonance frequencies. We believe that soft haptic devices will be an essential component in future developments of multifunctional electronic skin.

## 2 Experimental Section

### 2.1 Synthesis and Preparation of Conductive Composites

Initially, a distinct amount of silver (Ag) microflakes (Thermo Scientific, 99.9%, size: 4–8 μm) are dispersed in solutions of elastomer material based on a tri-block copolymer of poly(styrene-block-isoprene-block-styrene) (SIS, Sigma-Aldrich, 14 wt % styrene content) in toluene (Fisher Scientific, 99.5%)). Low adhesion substrates are formed by spin-coating polystyrene sulfonate (PSS) onto silicon wafers (5% (wt/vol) PSS:Na/deionized water solution, spin-coated at 2000 rpm). The Ag composite solutions are casted using doctor blade coating to form films of ∼200 μm thickness. The solid composite film is then immersed into a silver precursor solution consisting of various concentrations of silver trifluoroacetate (STFA, Thermo Scientific, 98%) and dry ethanol (Fisher Scientific, 99.9%). The notation STFA 20 refers to 0.2 g/mL STFA concentration. The composite material remains in the precursor solution for 45 min to allow for diffusion of organometallic precursor throughout the entire film. The wet composite film is dried in a vacuum desiccator prior to the nucleation of silver nanoparticles. The dry composite film is then sprayed with a reducing agent solution consisting of hydrazine hydrate (50% (v/v)), Sigma-Aldrich, 50%–60%), ethanol (25% (v/v)), and deionized water (25% (v/v)). This initiates Ag nanoparticle nucleation inside the composite film and consequently removes the sacrificial PSS layer. The resulting composite film, consisting of Ag microflakes and nanoparticles, is washed alternatively with ethanol and deionized water for 10 cycles to remove residual chemicals.

### 2.2 Fabrication of Soft Induction Coils (Figure S1)

The composite is transferred to a new PSS-coated silicon wafer and patterned into inductor coils (12 turns and 11 mm outer diameter) using a laser cutter (Trotec Speedy 300 flexx, fiber laser). A transfer polymer solution of poly(styrene-block-ethylene-block-butylene-block-styrene) (SEBS, Kraton polymers) and tetrahydrofuran (THF, Fisher Scientific, 99.9%) is casted on the coils. After solidification of the SEBS layer, coils are easily removed by introducing water from the edges of the composite and silicon wafer (dissolving the sacrificial PSS layer) and peeling the coil design without damaging the material. Two coils embedded in SEBS layers are faced toward each other to facilitate the double coil design. The electrical interconnection of these coils is achieved using vias in their centers, which is filled with a conductive paste consisting of SIS and Ag microflakes (30% (v/v)).

### 2.3 Fabrication of Soft Sensing Foams

The precursor solution is prepared by mixing cellulose nanofibrils (carboxymethylated CNF, 5 pass through microfluidizer, 1 wt %, Innventia AB), a conductive polymer dispersion of poly (3,4-ethylenedioxythiophene): polystyrene (PEDOT:PSS, Heraeus Clevios, PH1000, 1.3 wt % of PEDOT:PSS), and deionized(DI) water using a shear disperser (T 10 basic ULTRA-TURRAX) for 3 min at scale 5 speed. Next, using the same dispersing settings, the dispersion is mixed again after adding the crosslinking agent ((3-glycidyloxypropyl)trimethoxysilane, GOPS, Sigma-Aldrich, 98 wt %) according to the specified weight ratios. After vacuuming the solution for 5 min in a desiccator to eliminate bubbles, the dispersion is poured into a PDMS mold with an inner diameter of 10 mm and a thickness of 10 mm at the bottom for thermal insulation. The PDMS mold is prepared by adding a piece of Toray Carbon Fiber Paper (TGP-H-030) on the bottom, and after pouring the solution into the mold, a second piece is placed on the surface of the solution. A liquid nitrogen bath is used to freeze the mold without directly exposing the solution. To remove the frozen solution from the PDMS mold, the mold is cut, and the sample is dried in a freeze dryer (Benchtop Pro, SP Scientific) over a 24 h period. The foam is placed in an oven for 30 min at 140 °C for crosslinking, after which the foam is placed in a sealed glass crystallization dish next to a few drops of DMSO (dimethyl sulfoxide, Sigma-Aldrich, ReagentPlus, 99.5 wt %) that are not in direct contact with the foam. The foam is baked for 1 h at 80 °C followed by 2 h at 90 °C to remove residual DMSO. Two copper magnet wires (POLYSOL 155 1X0.05 MM HG, ELECTRISOLA) are connected to the carbon papers by conductive silver epoxy glue (MG Chemicals 8330S-21G), which is cured for 2 h at 65 °C.

### 2.4 Fabrication of Mechanically Soft Magnets

Soft magnets are formulated based on neodymium-iron-boron (NdFeB, MQFP-16-7, Magnequench, average diameter: 5 μm) microparticles and silicone elastomer (Ecoflex 00-10). To achieve permanent magnetization, one part of a two-component silicone elastomer is mixed with NdFeB microparticles and the magnetic putty is magnetized using a pulse magnetizer (Redcliffe 700-BSM) with magnetic fields exceeding 2.5 T. The magnetized putty is thoroughly mixed to ensure random magnetic orientation of the NdFeB microparticles, after which the putty is mixed with the second component of the silicone elastomer and poured into a coin mold (7 mm in diameter and 2 mm in thickness). During the curing process, magnetic particles are oriented in a specific direction by using a strong permanent magnet. Once the silicone elastomer is solidified, the coin-shaped deformable magnet is removed from the mold.

### 2.5 Assembly of the Soft Actuator

The sensing foam is fabricated by first placing two pieces of carbon paper (Toray Carbon Fiber Paper TGP-H-030, 2 mm in diameter) that are connected to two stretchable conducting wires in the bottom of the PDMS mold, after which the foam solution is dispensed into the mold (8 mm inner diameter and a 10 mm-thick insulating bottom). The foam fabrication process continues then as described earlier. The soft foam and magnet are glued together by a thin layer of a premixed solution of Ecoflex 00–10 (Smooth-On) under moderate heating (70 °C). Similarly, the magnet/foam unit is attached to the soft coil using an adhesive layer based on SEBS and THF solutions (10% (wt/vol)). The adhesive is applied to the coil and the sensing interconnect and the foam are placed while the THF solvent is still present in the SEBS film. The assembly is complete once the SEBS layer solidifies.

### 2.6 Characterization

Scanning electron microscopy (SEM) images are acquired using a Sigma 500 Gemini (Zeiss). Mechanical characterization is performed using custom-built stress–strain setup composed of LSQ300A-EO1 motorized stage and a force gauge (M5-2, Mark-10). Tensile stress–strain experiments were performed with a deformation rate of 1% strain s^–1^. Characterization of the electrical properties of the stretchable conductor composites is performed using a Keithley 2400 source-meter in 4-point probe geometry with an interprobe distance of 2 mm (sample geometry: 3 cm × 0.5 cm × 200 μm (l × w × h)). The electromechanical measurements are performed using a custom-built system composed of an LSQ300A-EO1 motorized stage and a Keithley 2701 Ethernet Multimeter data acquisition system with mechanical clamps having 2 probes on each side (sample geometry: 3 cm × 0.5 cm × 200 μm (*l* × *w* × *h*)). Electromechanical measurements are carried out with a tensile deformation rate of 1% strain s^–1^. Magnetic measurements of soft coils are performed by coupling an AC/DC magnetometer (Extech SDL900) to a motorized stage (LSQ300A-EO1). The spatial resolution of the magnetic measurements is 50 μm. During magnetic and magnetic force measurements, soft coils are powered using a high current source meter (HP-E3631A). Infrared images of soft inductor coils under operation are acquired using a thermal camera (FLIR A320-G). Force measurements of soft haptic devices at different DC excitation currents supplied by a power source (Keithley 2230G-30-6) are acquired using a force gauge (M5-012, Mark-10), while the soft magnet is fixed in displacement. The vibration amplitude spectrum is measured in pulse excitation mode using a computer-controlled source-measurement unit (Keithley 2612B) which supplies 1 A excitation current to the actuator at different frequencies ranging from 10 to 130 Hz while measuring the foam resistance at 300 Hz. As the sensor operates in compression mode, the minimum resistances are averaged for each actuation frequency, and the difference between the base resistance and the average minimum resistance is plotted.

### 2.7 Laser Interferometry

To measure the displacement of the actuator, we used a custom laser heterodyne interferometer.^35^ In this device, the beam of a frequency-stabilized helium-neon laser was projected toward the actuator using a 25× lens with a numerical aperture of 0.4. The light reflected back from the actuator traveled through the objective lens to a Faraday rotator that allowed forward-propagating light to be separated from reflected light. The reflected light was then made to interfere with a reference beam on a pair of photomultipliers. The output from the photomultipliers was fed to a differential amplifier and sampled by an A/D board. Custom LabVIEW software was used to calculate the displacement from the sampled reference and object signals, using the well-known arctan method. Calibrations of the device showed that displacements between 0.1 and 1000 nm were faithfully detected over the frequency range between 5 and 3000 Hz (limitations in the strain-gauge sensor used as the reference precluded calibration measurements at frequencies exceeding 3 kHz). The noise floor of the device is approximately constant at 15 pm/sqrt (Hz) between 100 Hz and 50 kHz. When measuring its displacements, the actuator was driven by a 33220A function generator (Agilent Technologies) with 50 Ohms output impedance. Using an 8 mA sine wave excitation current, the actuator is driven at frequencies ranging from 10 to 200 Hz while measuring the actuator movement using laser interferometry. The amplitude of the response is averaged over the 873 ms excitation time.

## 3 Results and Discussion

One critical component of our soft electromagnetic actuators is the intrinsically stretchable conductor for the deformable coils. To generate soft electrodes that can sustain electrical currents reaching several amperes under mechanical deformation, we developed a concept around in situ formation of AgNPs in elastomer-AgF nanocomposites (Figure 1b). We employed poly(styrene-block-isoprene-block-styrene) (SIS) as the elastomer matrix and mixed it with AgFs in the solution to cast the initial composite conductor on glass wafers (Figure 1b–d). The initial composite was immersed in an ethanol-silver precursor solution (silver trifluoroacetate, STFA) to initiate controlled growth of nanoparticles on the SIS backbone, which possess an unsaturated double bond that is ideal for the reduction of silver ions (Figure 1b,f–h).^32^ The structural characterization of the initial form of the conductive composite performed using scanning electron microscopy (SEM) exhibits simply AgFs dispersed in the SIS matrix (Figure 1c,d). The Ag flakes organize in a nacre-like structure at the surface of the conductive composite (Figure 1c), which is also supported by the cross-section SEM image of the initial form of the conductive composites (Figure 1d). This nacre-like organization is the key to reaching high electrical conductivity values with a low volumetric fraction of the filler material.^36,37^ The AgNPs are introduced to the composites by immersing various concentrations of STFA solutions (notation STFA 20 corresponds to 0.2 g/mL STFA), and subsequently reducing the silver ions by hydrazine treatment (Figure 1b,e,f). The concentration of STFA solutions controls the volumetric concentration of the Ag nanoparticles in the system, which differentiates this conductive composite from earlier studies performed on AgF/AgNP composite systems.^21^ The structural characterization of the conductive composite after nanoparticle nucleation clearly shows the formation of Ag nanoparticles both at the surface of silver flakes and in the SIS elastomer matrix (Figure 1e–j). The surface and the cross-section SEM images of the AgF/AgNP composite highlight a nacre-like structure analogous to the initial form of the conductive composite with the only difference being the presence of Ag nanoparticles (Figure 1e inset). The higher magnification SEM images of the AgF/AgNP conductive composites indicate that the NP growth in the polymer matrix occurs in a random fashion, as the image depicts the random distribution of nanoparticles in the SIS matrix (Figure 1f). To assess the nanoparticle growth in these composites, we also characterized the structure of the nanocomposites processed using higher concentrations of STFA solutions (Figure 1g–j). SEM images suggest that increasing the concentration of STFA solutions leads to the formation of nanoparticle clusters consisting mainly of nanoparticles with larger dimensions (Figure 1h). By further increasing the STFA concentration, these clusters merge and generate Ag nanoparticles exceeding 100 nm in size (Figure 1i,j). These structural modifications triggered by the high Ag salt concentration influence the electrical and mechanical properties of conductive composites becoming highly conducting but brittle.^33^ A combinatorial analysis of the mechanical and electrical properties of the SIS/AgF/AgNP composite system is needed to optimize the performance of the stretchable electrodes for specific purposes.

**Figure 2:**
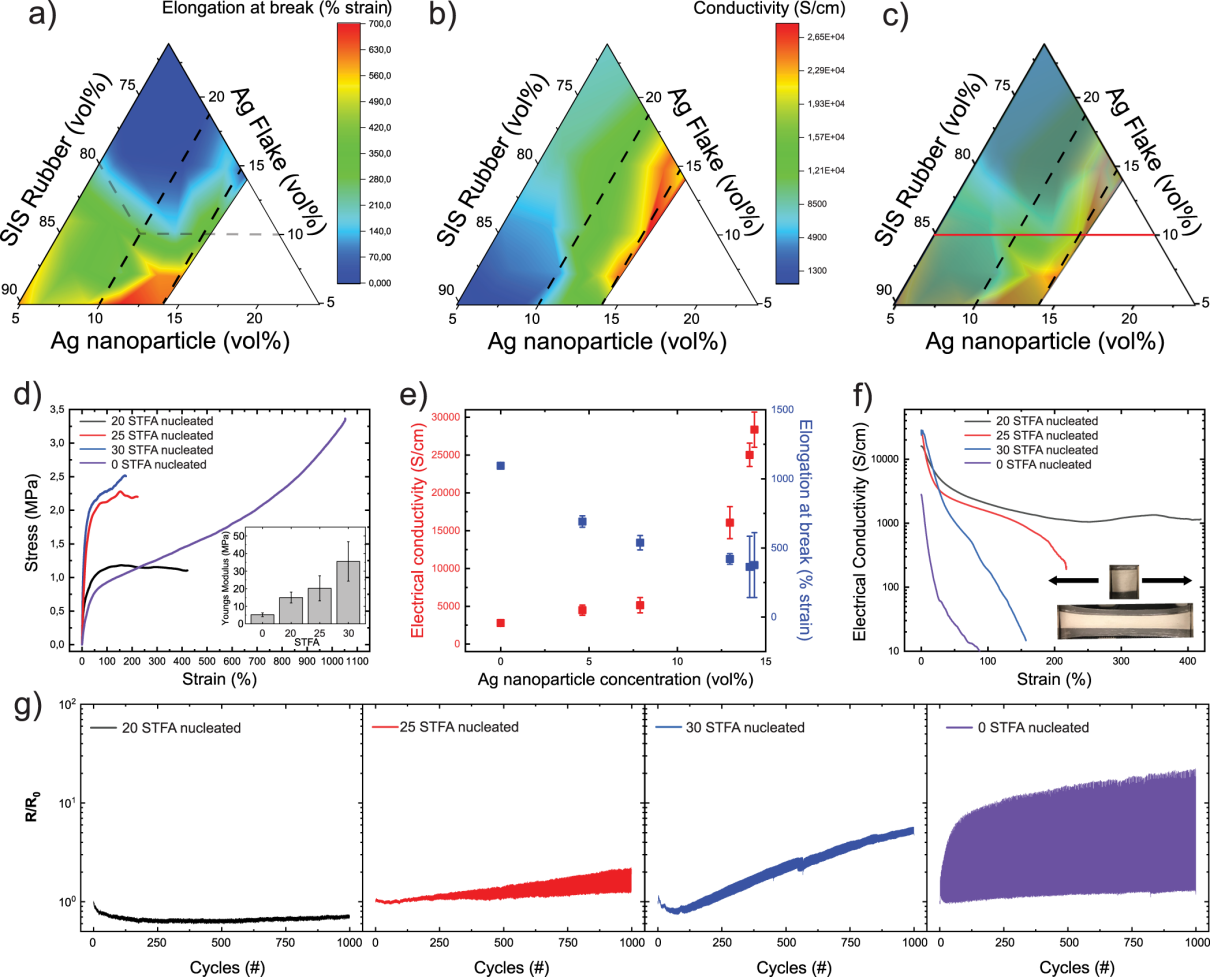
Performance of the stretchable conductors. Ternary density plots highlight the influence of composition of stretchable conductors on (a) elongation at break and (b) electrical conductivity. (c) Composite ternary density plot consisting of overlay of ternary density plots for elongation at break and conductivity. (d) Stress–strain curves of composites of varying AgNP content with inset showing Young’s modulus. (e) Electrical conductivity and elongation at break for composites as a function of AgNP concentration. (f) Conductivity vs strain for composites during singular mechanical failure test (images of stretchable electrodes with 10% (v/v) Ag flake, STFA 20 prior and after tensile deformation at 420% tensile strain). (g) Strain cycling at 20% strain for various composite formulations.

The combinatorial study on the mechanical and electrical properties of the conductive composites was performed using 36 composite samples of various volumetric concentrations in SIS, AgFs, and AgNPs. The results are summarized in ternary phase diagrams reporting the elongation at break and electrical conductivity as the critical parameters illustrating their mechanical and electrical properties, respectively (Figure 2a,b). The concentration of grown AgNPs was estimated from the dry mass increase of the samples, with 13% (v/v) AgNPs for STFA 20, 14.1% (v/v) AgNPs for STFA 25, and 14.5% (v/v) AgNPs for STFA 30. In this composite system, it was not possible to exceed AgNP concentrations higher than 14.5% (v/v) (Figure 2a,b). The ternary phase diagram for elongation at break reveals that a high concentration of AgFs makes the composite brittle, while a high concentration of AgNP tends to soften the composite (Figure 2a). This softening is probably due to the plasticizing effect of the STFA that was trapped in the composites^33^ and leads to a strain of up to 700%. The ternary phase diagram mapping the electrical conductivity of the composites indicates that composites with high content in AgFs exhibit high electrical conductivity (Figure 2b). This effect is further amplified by the incorporation of AgNPs such that the highest electrical conductivity values (∼25,000 S/cm) are observed in composites with high AgNP content and high overall filler fraction (Figure 2b). To find an ideal composition from these phase diagrams that take both mechanical and electrical aspects of the composites into consideration, we prepared a composite phase diagram by overlaying both (Figure 2c). We identified a region of interest to explore further at 10% (v/v) AgFs and varying AgNP content. This region is outlined by concentration lines of AgFs (10% (v/v)) and AgNPs 10% (v/v) and 14.5% (v/v), and it corresponds to composites of both remarkable stretchability and electrical conductivity. To establish the ideal AgNP concentration in this region, we studied the mechanical and electrical properties of composites with different AgNP concentrations (Figure 2d–g). Increasing AgNP content results in lower elongation at break and higher Young’s modulus (Figure 2d), while the initial electrical conductivity increases rapidly for AgNP concentrations >10% (v/v) (Figure 2e). At the highest AgNP concentrations, the variation in electrical conductivity and elongation at break between samples increases substantially, indicating that the composite becomes less homogeneous and potentially less robust (Figure 2e). The electromechanical characteristics of these conductive composites are evaluated using electrical failure and cyclic deformation experiments (Figure 2f,g). The absence of AgNPs in the conductive composites prompts a high strain dependence in electrical conductivity (Figure 2f). Higher concentrations of AgNPs allow the composites to sustain their electrical conductivity above 4600 S/cm at tensile strain values up to 20% (Figure 2f). The composite with 13% (v/v) AgNPs maintains an electrical conductivity of >2000 S/cm up to 100% tensile strain, which makes it the most strain-stable conductor among the compared compositions (Figure 2f). This composite can be stretched up to 420% tensile strain, while still exhibiting an electrical conductivity of 1100 S/cm, before mechanical failure (Figure 2f). Cycling the deformation to 20% strain showed that the change in relative resistance was very low for the 13% (v/v) AgNP composite, while both higher AgNP loading and no AgNPs resulted in a stronger strain dependence (Figure 2g). The addition of moderate concentrations of AgNPs can improve the percolation network of the AgFs by bridging the gaps between the flakes. The addition of the STFA is also plasticizing the composite and affects the electromechanical properties. Too much nucleation of AgNPs, however, creates larger interconnected AgNP clusters, which makes the composite more brittle and less stable during strain cycling. Strain cycling to high strains gives the same trend in relative performance (Figure S2). This degradation of electromechanical properties may result from delamination as well as microcrack generation and propagation due to the repeated mechanical loading on the soft conducting composite.^21^ Altogether, the composite with 10% (v/v) AgFs and 13% (v/v) AgNPs showed the best combined mechanical and electrical properties and was chosen as the base material for the fabrication of soft electromagnetic coils.

**Figure 3:**
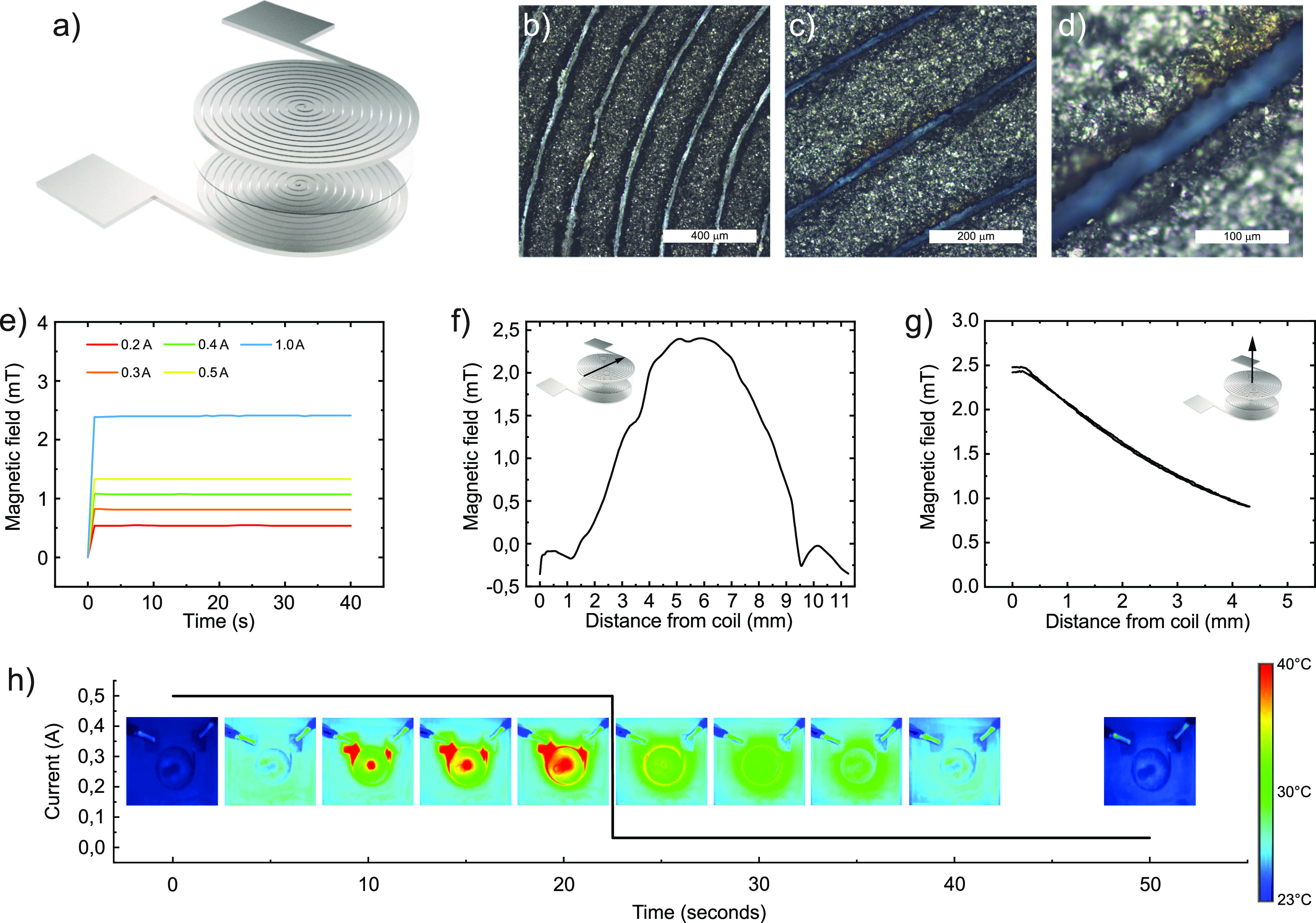
Fabrication and characterization of soft coils. (a) Schematic illustration of a double-layer soft electromagnetic induction coil. (b–d) Microscope images of a patterned soft coil at different magnifications. (e) Temporal evolution of the magnetic field of soft electromagnetic induction coils under various excitation currents. (f) In-plane and (g) out-of-plane spatial distribution of the magnetic field of soft induction coil at 1 A continuous excitation. (h) Heat maps of the soft electromagnetic coil during and after excitation of 0.5 A continuous current.

Soft electromagnetic coils consisting of 20 turns were laser fabricated from the developed and optimized conductor (Figures 3a and S1). To enhance the generated magnetic field, two coils were stacked and interconnected in the middle to establish a double-layered coil structure with external contacts (Figure 3a). The fiber laser cutting of the 200 μm-thick composite film produced regular patterns with only 30 μm separation between each turn of the coil (Figure 3b–d). The patterned soft coils are transferred onto a silicone substrate and folded as pairs with a SEBS separation layer to form double-layered coils (Figures 3a and S1). The highly conductive coils exhibit electrical resistance as low as 1.5 Ω (Figure S4) and could generate stable magnetic fields reaching >2 mT under 1 A excitation (Figure 3e), while operation currents exceeding 1 A resulted in drift in the magnetic fields, likely originating from local effects caused by joule heating. The magnetic field distribution was characterized by in-plane and out-of-plane displacements with respect to the coil center surface (Figure 3g,h). The magnetic field strength is preserved within a circle of 4 mm diameter measured from the center (Figure 3f). The soft coils sustained 75% [40%] of their peak field strength at out-of-plane displacements of 2 mm [4 mm] (Figure 3g). To identify the heat generated by the soft inductor coils under operation, we employed thermal imaging (Figure 3h). This characterization revealed that operational currents of 0.5 A led to a temperature below 35 °C in the coil and 40 °C at the contact points (Figure 3h). Higher currents up to 2 A can be achieved but the coil becomes too hot to be in direct contact with the skin (Figure S3), such that the safe current range is below 1 A. The spontaneous cooling behavior after operation highlights that these soft inductors can dissipate heat effectively likely due to the high surface area of the pattern and the decent thermal conductivity of the composite. The devices returned to the initial temperature in 30 s after operation for all currents (Figures 3h and S3). Hot spots are visible at the via in the center of the coil and at the contacts, likely due to high local resistance. Hot spots may be avoided by widening the via and the contacts, and overall heating can be reduced by improving the heat dissipation by using thinner encapsulation with higher heat conduction.

**Figure 4:**
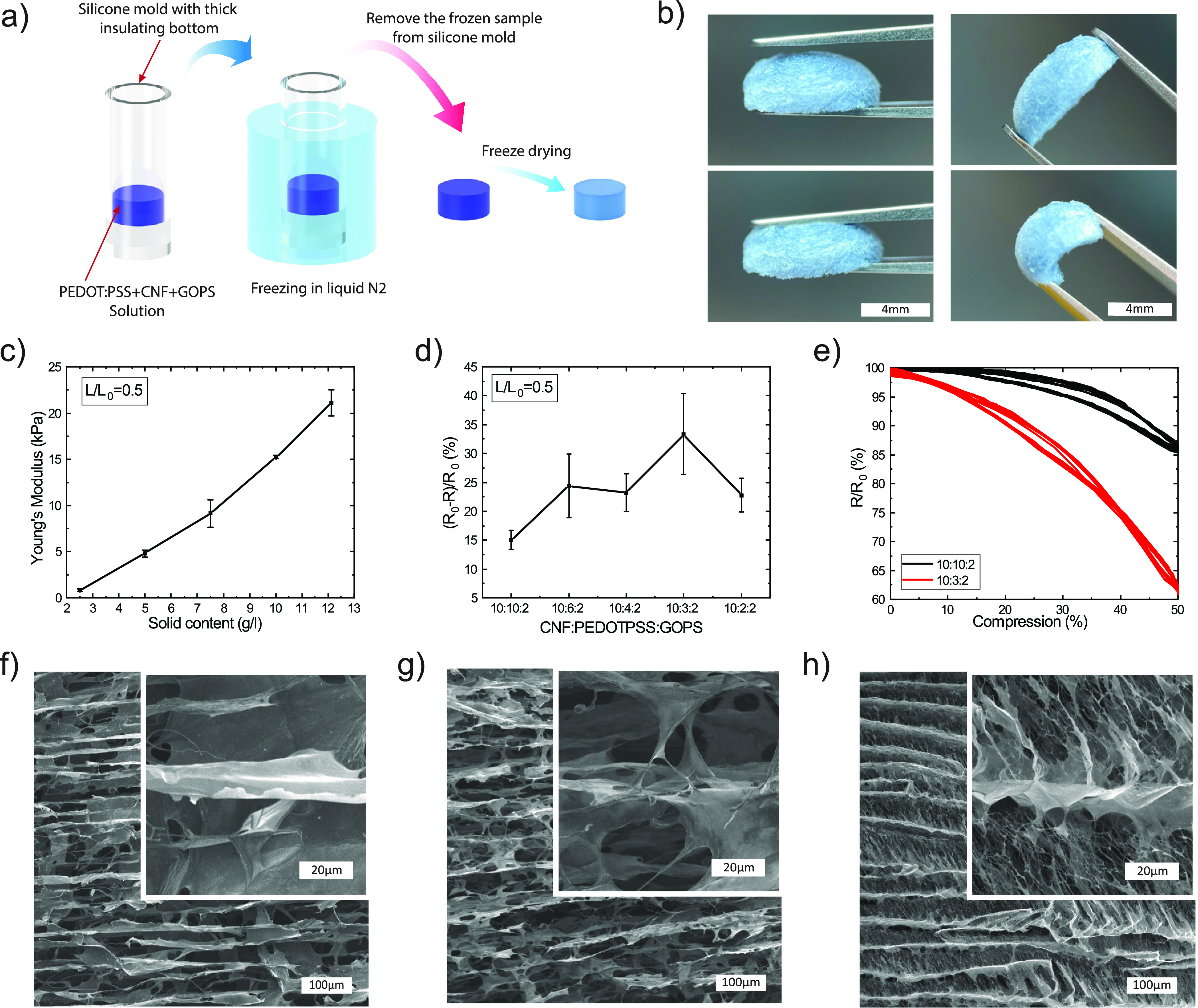
Compressible sensing foams. (a) Schematic of the fabrication process for the soft cellulose-based sensing foam. The foam is made from a dispersion comprising PEDOT:PSS, cellulose nanofibrils (CNFs) and GOPS. The solution was dispensed into a thick-bottomed PDMS mold, then immersed in liquid nitrogen to flash freeze the solution, after which the frozen foam was freeze dried in vacuum. (b) Photograph of the resulting sensing foam. (c) Young’s modulus at 50% compression for foams with varying total solid content. (d) Change in foam resistance response to 50% compression when varying the ratio of PEDOTPSS in the 2.5 g/L foam precursor solution. (e) Sensor relative resistance change as a result of compressing the foam to 50% of its initial length for two different PEDOT:PSS weight ratios. (f) SEM image of the initial 12.1 g/L solid content foam at 10:10:2 (CNF:PEDOT-PSS:GOPS) weight ratio. (g) SEM image of the 2.5 g/L solid content foam at 10:10:2 weight ratio. (h) SEM image of the 2.5 g/L solid content foam with less PEDOT:PSS (10:3:2 weight ratio).

The next part of the resonator is the soft foam, which separates the coil from the soft magnet. By developing and incorporating a soft compressible strain-sensing foam, we aimed at integrating amplitude-sensing capability into the resonator. Compressive strain-sensing can be achieved by utilizing conductive cellulose-based foams,^38^ which can be compressible, pressure-sensitive, and lightweight. The cellulose nanofibrils (CNF) provide a structural template for the self-assembly of the conductive polymer (PEDOT:PSS); GOPS acts as the crosslinker for PEDOT:PSS which makes the foam more elastic and stable. The foam fabrication process is presented schematically in Figure 4a. Foams fabricated according to a previously reported recipe^38^ had Young’s modulus of around 30 kPa (at 30% compression), which is too stiff for the intended application. Two approaches were combined to soften the foams: directional freezing and lowering of solid content. By using a cylindrical PDMS mold with thick insulating bottom, radial cooling of the solution was achieved. This gives ice crystal formation in the direction of the heat transfer,^39^ which induced an anisotropic foam structure which is softer in the axial direction. The fabricated foams were lightweight, compressible, and flexible (Figure 4b). By lowering the solid content in the anisotropic foam, it became even softer and lighter (Figure 4c), and Young’s modulus of around 1 kPa was achieved by lowering the solid content from 12.1 to 2.5 g/L. Further reduction of solid content was limited by increasing shrinkage of the foam after drying. The sensing capability of the foams was tested by compressing them to 50% of their initial length and measuring the difference in resistance. The resistance of 2.5 g/L foam changed by approximately 15% when it was compressed by 50%. By changing the PEDOT:PSS content of the foam, it was possible to tune the conductor percolation network and thereby increase the strain sensitivity of the foam (Figure 4d). The resistance sensitivity of the conductive foams under 50% compression in the axial direction increased from 15 to 33% by decreasing the amount of conductive polymer in the foam (by changing the precursor solution weight ratios from 10:10:2 to 10:3:2). The foams show little hysteresis under compression cycles and the improvement in sensitivity for the low PEDOT:PSS foams is present over the full strain range (Figure 4e). The foam’s sensitivity to temperature and Young’s modulus also change with the conductive polymer content (Figure S5a,b), but this effects are relatively small. SEM imaging of the foam (12.1 g/L) cross-section shows an anisotropic structural configuration induced by the direction of heat transfer in the freezing process (Figure 4f). This is in line with the observation that the foam is more compressible in the axial directions than in the radial direction. When solid content decreased to 2.5 g/L the cavities between layers became larger and the layers became less dense, which resulted in softer foams (Figure 4g). Lowering of the PEDOT:PSS content in the 2.5 g/L foam had a pronounced effect as it induced fibrillar structures in between the layers in the foam (Figure 4h). This structural morphology can be one of the explanations for the improved electrical sensitivity to compression for this foam, as the fibrillar structures can improve the connectivity between the sheet layers during compression.

**Figure 5:**
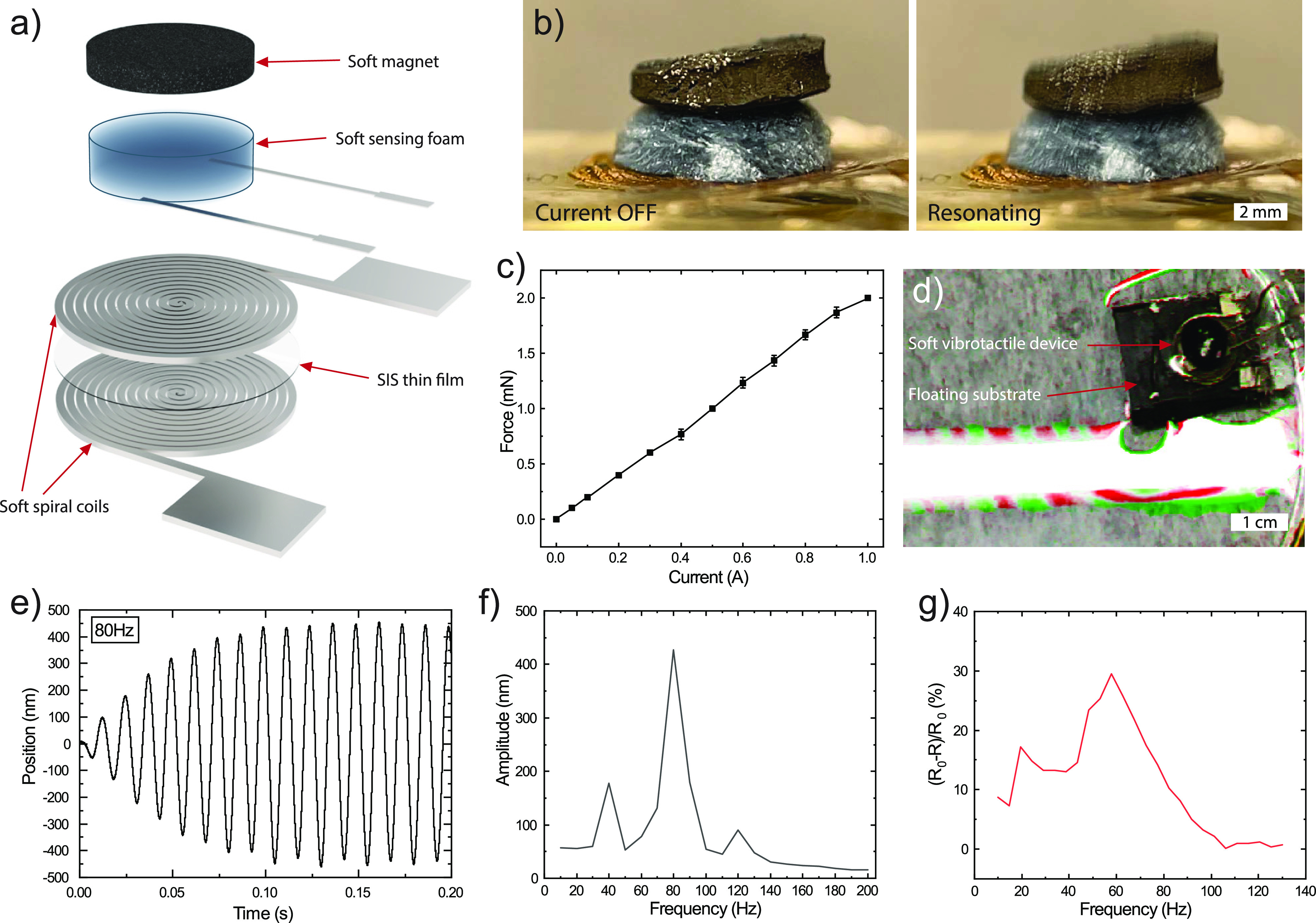
Soft haptic device with integrated sensing. (a) Schematic illustration of soft electromagnetic haptic device that includes two stacked spiral coils with an SIS spacer film, a soft cellulose-based sensing foam which is connected using two soft silver composite conductors for monitoring, and a soft NdFeB-silicone composite magnet. (b) Assembled device in relaxed and resonating states. (c) Generated force on the soft magnet by the coil at different DC excitation currents at fixed displacement corresponding to the actuator geometry. (d) Soft electromagnetic haptic device generated waves on the water’s surface when actuated in 10 Hz pulsing mode (difference in video frames visualized in colors). (e) Laser interferometry measurements of the device’s actuation while being excited by 8 mA sine waves at 80 Hz. (f) Vibration amplitude vs excitation frequency measured with laser interferometry (8 mA excitation). (g) Relative change in the resistance measured with integrated sensing cellulose-based foam while the device is being excited by 1 A pulse excitation at different frequencies.

By integrating the developed coils and the sensing foams with an elastomer-based composite magnet, a soft vibrotactile actuator was achieved (Figure 5a,b). The soft magnet consisted of magnetized NdFeB microparticles dispersed in soft Ecoflex silicone rubber. During curing, an external magnetic field was applied to align the magnetic particles. The compression sensor signal was red out by two AgF composite conductors connected to the bottom of the sensing foam. The device actuation was tested by applying an excitation current of 1 A at 40 Hz which resulted in the vibration movement of the actuator of approximately 200 μm in amplitude (Figure 5b). The vibration amplitude is directly related to the force generated on the soft magnet, which was measured for various DC excitation currents when the soft magnet displacement was fixed (Figure 5c). At 1 A, a force of 2 mN was reached, which corresponds to the weight of 200 mg. The vibrations generated by the actuator when lying on a table can be clearly felt and heard. To visualize the vibration of the device, the soft actuator was placed on a hydrophobic carbon paper to allow it to float in a water bath. When activated, the vibrations emitted from the device generated waves that were clearly visible in the water (Figure 5d and Movie S1). For vibrotactile actuators, the vibration amplitude can depend strongly on the excitation frequency due to various vibration modes. This is shown in Movie S2, which illustrates the device’s performance at frequencies ranging from 10 to 100 Hz. We therefore measured the amplitude response of our actuator using confocal laser interferometry^35^ (Figure 5e,f). The amplitude response showed a major resonance peak at around 80 Hz (the peak amplitude at this frequency was 426 nm), and resonance peaks of smaller amplitude at 40 and 120 Hz. A comparison of the excitation current and the device’s response at 10, 40, 80, and 120 Hz measured by this method is shown in Figure S6, which illustrates how excitation at resonance frequencies can result in a significant increase in the vibration amplitude. When the vibration amplitude instead was measured with the integrated sensing foam in a similar device, a major resonance peak was detected around 60 Hz, and a minor one at 20 Hz (Figure 5g). There are four known end organs in the glabrous skin that perceive different vibration ranges: the Merkel Disk is responsive to vibration at below 5 Hz, Meissner Corpuscle (from 3 to 100 Hz), Ruffini Ending (15 to 400 Hz), and Pacinian Corpuscle (10 to 500 Hz).^40^ Our developed actuators operate at relevant frequencies for three of these end organs. The resonance frequency of the actuator can be tuned by changing the soft magnet mass or the soft foam thickness, or by adjusting the stiffness of the foam according to Figure 4c. These parameters explain the difference in resonance frequency between two similar devices in Figure 5f,g. The actuation resonance frequency can also be determined by the stiffness of the contacting substrate. Therefore, for effective vibrotactile actuation, vibration amplitude sensing is required so that the device’s actuation frequency matches the system’s resonance frequency. The small size of the device makes it attractive for on-skin applications. Further miniaturization is currently limited by insufficient force generation and laser patterning resolution, however, further improvement in force generation by decreasing the distance between coil and magnet and by using a stronger magnet would facilitate miniaturization. The device stack is designed in a way that it promotes deformations, as the ultrasoft foam can buffer deformations between the soft coil and magnet.

## 4 Conclusions

Soft vibrotactile devices have the potential to expand the functionality of emerging electronic skin technologies. Here, we have worked toward that goal by developing a new kind of soft vibrotactile device with integrated vibration sensing. Several materials and components of the device were developed: (i) high-performance soft and stretchable conductors were needed to supply the high currents necessary for electromagnetic actuation. A new concept around controlled in situ AgNP formation was developed which improved the conductivity five times with respect to pure AgF conductors. (ii) High-aspect-ratio conductor structures were required for the coils. A laser patterning approach was developed which yielded 200 μm-thick conductor lines with only 30–50 μm separation. (iii) To tune the vibration frequency and enable vibration amplitude sensing, soft cellulose-based sensing foams were developed. By tuning the composition of the foams, ultra-soft and lightweight foams with electrical sensitivity to compression were achieved. The above components together with a soft magnet were finally assembled into soft vibrotactile devices. The vibration spectrum for various frequency excitation was characterized both with external measurement equipment and the internal integrated sensing foam. The incorporation of additional functional materials in the soft foams, e.g., 2D materials,^41^ may further improve the performance. Altogether, we have demonstrated how various material concepts can be joined together to yield a new kind of vibrotactile device. We believe that this will contribute to the development of more sophisticated soft haptic devices which will be an integrated feature in future electronic skin and wearable applications.

## Supporting Information Available

Fabrication schematic, soft conductor performance under cyclic tensile deformation, thermal characterization of soft electromagnetic coils during and after excitation, resistance measurements of soft inductor coils, influence of humidity and composition on foam performance, excitation current and responses of the vibrotactile device using laser interferometry (PDF)
Soft electromagnetic vibrotactile actuator operating at different frequencies (MP4)
Actuation of floating soft electromagnetic vibrotactile device (MP4)
