# Supplementary material for: Soft Electromagnetic Vibrotactile Actuators with Integrated Vibration Amplitude Sensing
Source: ACS Appl Mater Interfaces. 2023 Jun 16;15(25):30653–62. doi: 10.1021/acsami.3c05045 (PMC10316331; doi:10.1021/acsami.3c05045)
Supplement: Supplementary file 1 — am3c05045_si_001.pdf [file am3c05045_si_001.pdf]

SUPPORTING INFORMATION

Soft electromagnetic vibrotactile actuators with integrated vibration amplitude sensing

Mert Vural,[1,2‡] Mohsen Mohammadi,[1,2‡] Laura Seufert,[1] Shaobo Han,[1] Xavier Crispin,[1,2] Anders Fridberger,[3] Magnus Berggren,[1,2] and Klas Tybrandt[1,2]*

[1] Laboratory of Organic Electronics, Department of Science and Technology, Linköping University, 602 21 Norrköping, Sweden.

[2] Wallenberg Wood Science Center, ITN, Linköping University, Norrköping, Sweden.

[3] Department of Biomedical and Clinical Sciences, Linköping University, Linköping, Sweden.

[*]Email: klas.tybrandt@liu.se

[‡] Equal contributions

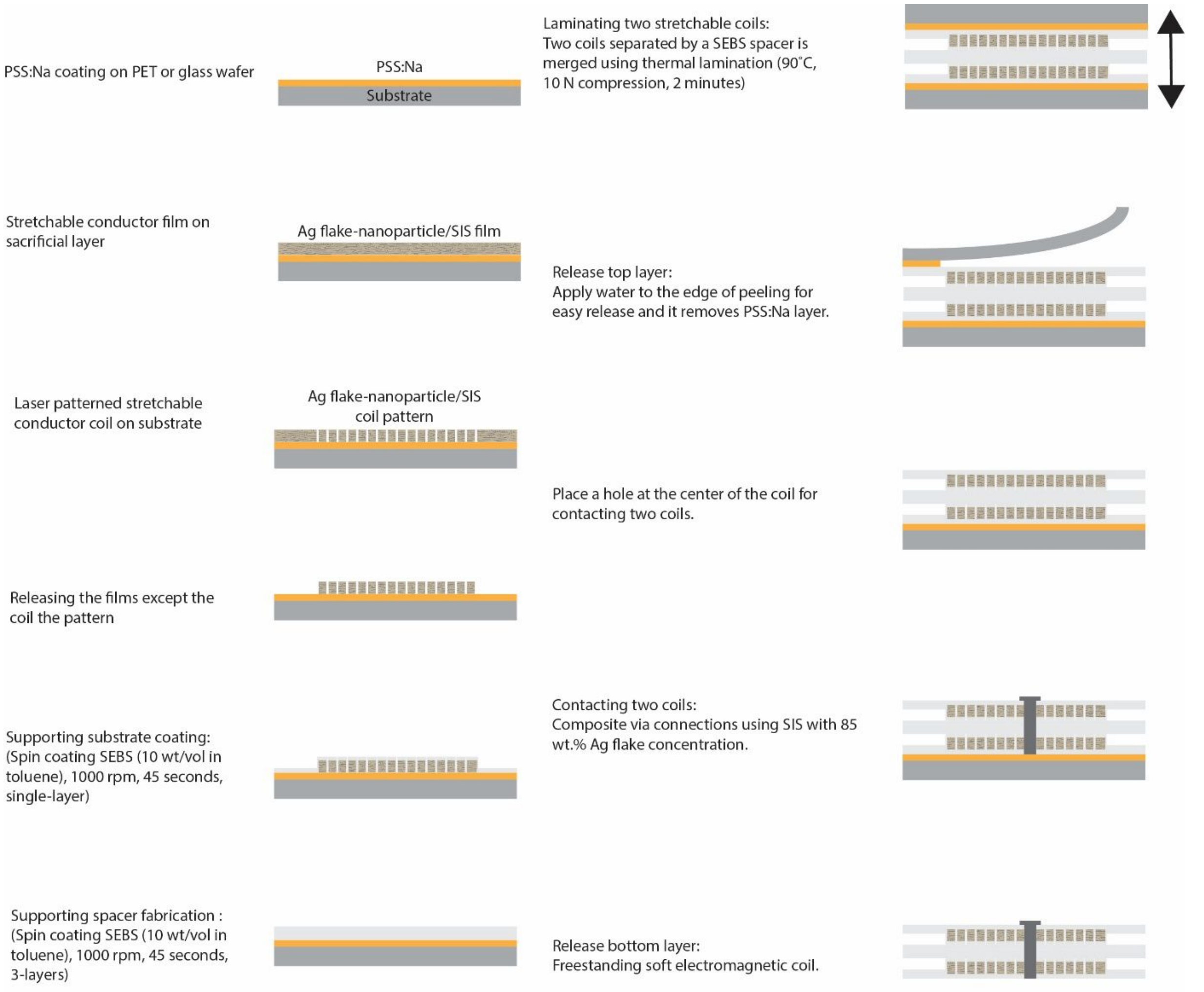

Figure S1. Fabrication protocol for single and double stretchable inductor coils. First a water soluble sacrificial layer of PSS:Na is spin coated on glass wafers. This step is followed by coating of an AgF/SIS (10% (v/v)) composite film using a doctor-blade. The coated film is dried overnight in ambient conditions to remove excess solvent. The dried film is immersed in AgNP precursor solution with 20% (wt/vol) silver trifluoroacetate solution (STFA) for 45 minutes and then

nucleated using a reducing agent. The resultant composite film is washed with water and ethanol to remove excess precursor, reducing agent and reaction products. The composite film is then dried in vacuum overnight. The AgF/AgNP/SIS composite is fashioned into coils using a laser cutter. The excess material is removed using the sacrificial layer. Single inductor coils are covered with an elastomeric material (SEBS) using spin coating to facilitate a base for transfer. To generate a robust double coil actuator, a spacer substrate is prepared by spin coating SEBS on PSS:Na coated glass wafers. This spacer is used to attach two separate coils to each other using thermal lamination. The merged double coils are removed from their respective substrates by simply applying water at the edges of the wafer, which simply dissolves sacrificial PSS layers and release coils from the glass wafer substrates. The coils are electrically connected by placing a via connection through their center, which is filled with a high concentrated AgF/SIS composite (85 wt.%, ~30% v/v). After introducing the via connections, the stretchable double coil is packaged using SEBS substrates with exposed electrodes to facilitate actuation.

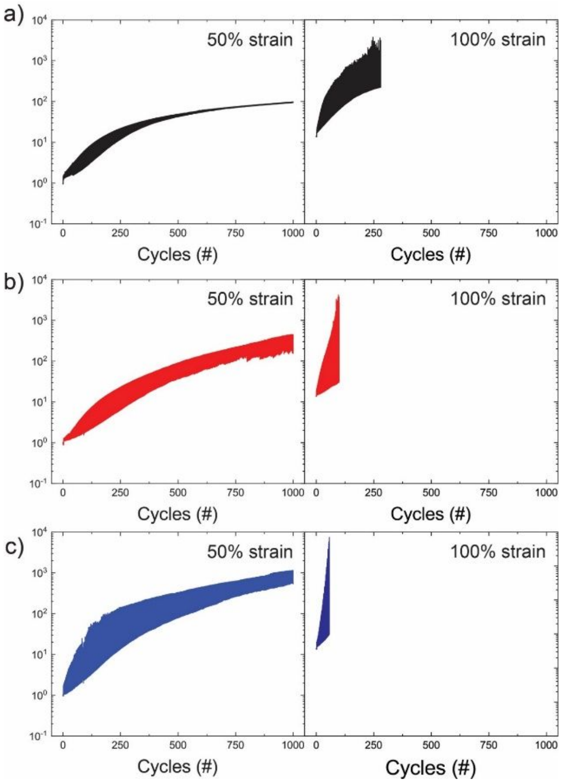

Figure S2. Relative resistance change (R/R_0) for soft conductors with 10% (v/v) AgF incubated in a) STFA 20, b) STFA 25, c) STFA 30 during cyclic (50% and 100% strain) tensile deformation.

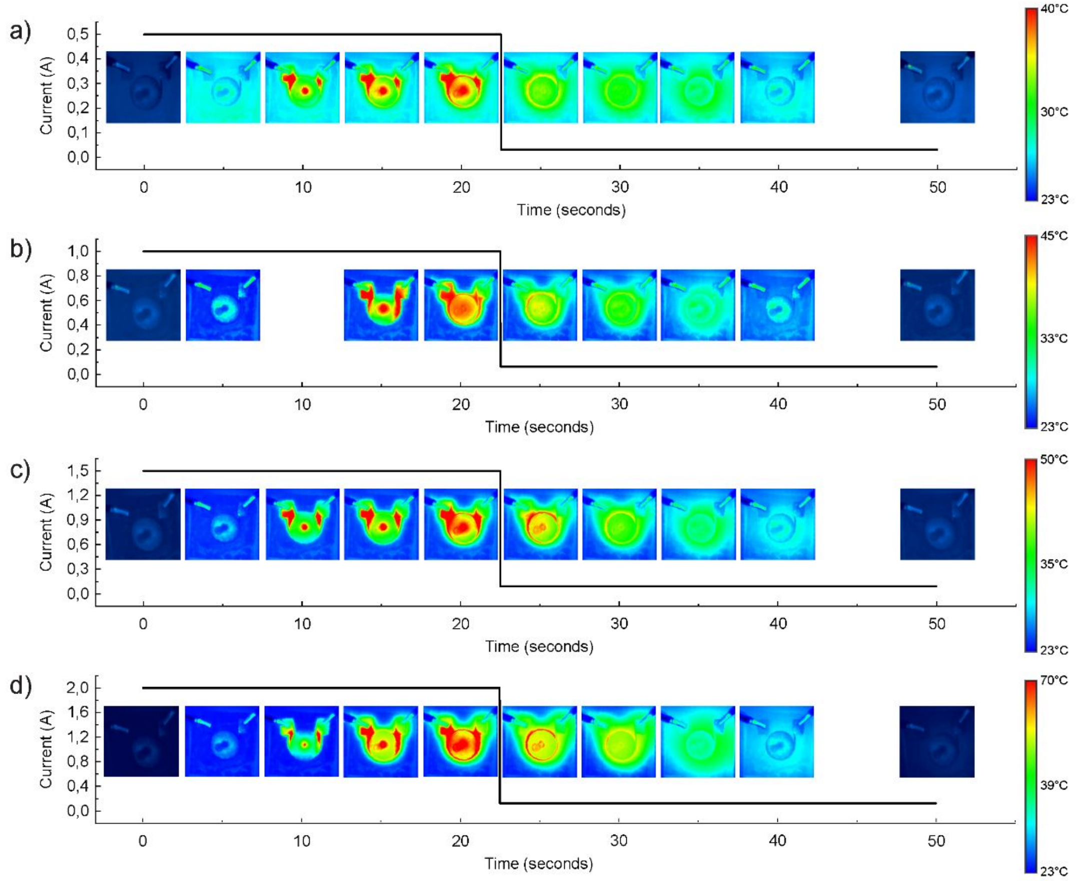

Figure S3. a) Heat maps of soft electromagnetic coils during and after excitation under continuous currents of i) 0.5A, j) 1A, k) 1.5A, l) 2A.

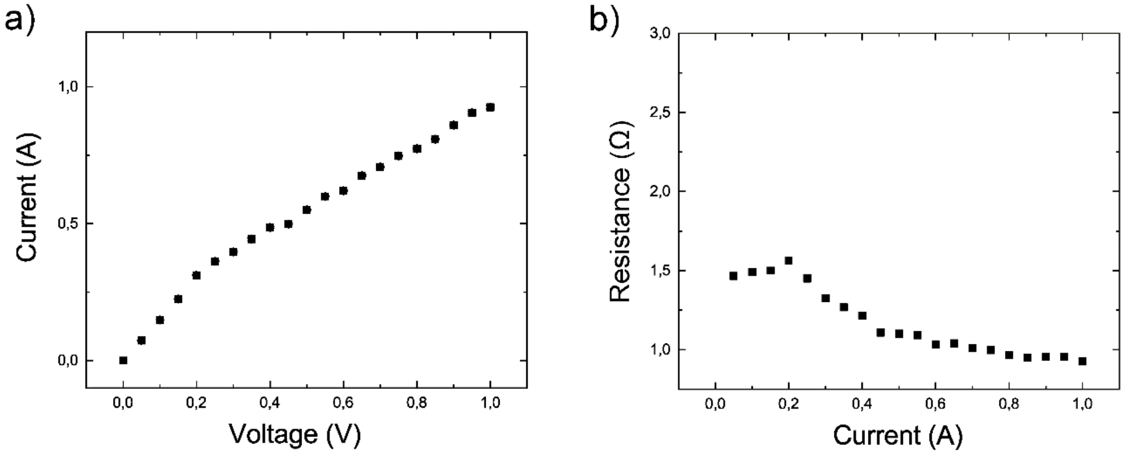

Figure S4. a) I-V curves of soft inductor double coil assembly. b) Change in resistance of soft inductor coil assembly as a function of excitation currents. The resistance of soft double coils decreases slightly with increasing current, which could originate from local sintering of particles into conductive pathways.

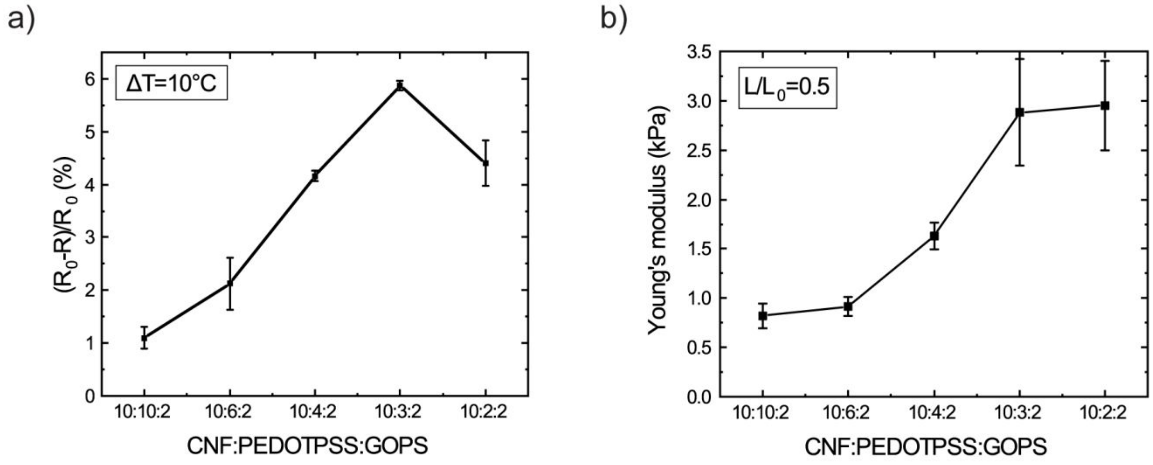

Figure S5. a) Change in foam resistance response to 10 K increase in temperature. b) Change in foam Young's modulus at 50% compression for varying ratios of PEDOTPSS in the 2.5 g/l foam precursor solution.

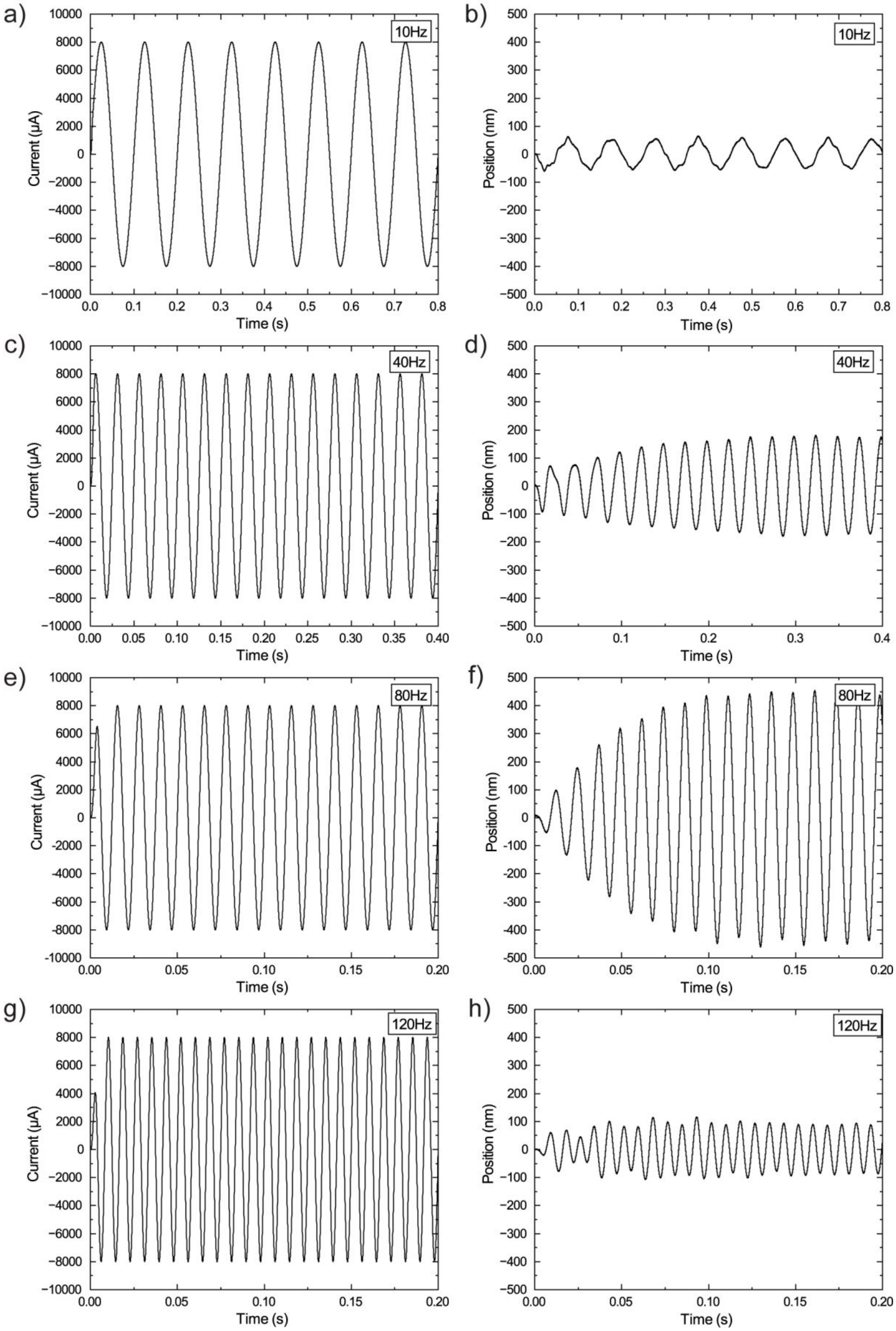

Figure S6. The excitation current and laser interferometry measurements of the device's actuation

at a,b) 10 Hz, c,d) 40 Hz, e,f) 80 Hz, and g,h) 120 Hz.
